# Supplementary material for: Usability of machine learning algorithms based on electronic health records for the prediction of acute kidney injury and transition to acute kidney disease: A proof of concept study
Source: PLoS One. 2025 Jul 1;20(7):e0326124. doi: 10.1371/journal.pone.0326124 (PMC12212546; doi:10.1371/journal.pone.0326124)
Supplement: S1 File — (DOCX) [file pone.0326124.s001.docx]

**Usability of Machine Learning Algorithms based on Electronic Health Records for the Prediction of Acute Kidney Injury and Transition to Acute Kidney Disease: a Proof of Concept Study**

Lorenzo Ruinelli^1,2^, Pietro Cippà^3,4^, Chantal Sieber^3^, Clelia Di Serio^2^, Paolo Ferrari^5,6^ and Antonio Bellasi^3,4^

1. Area ICT, Ente Ospedaliero Cantonale, 6500 Bellinzona
2. Clinical Trial Unit, Ente Ospedaliero Cantonale, 6500 Bellinzona
3. Division of Nephrology, Ente Ospedaliero Cantonale, 6900 Lugano
4. Faculty of Biomedical Sciences, Università della Svizzera italiana, 6900, Lugano, Switzerland
5. Department of Medicine, Ente Ospedaliero Cantonale, 6500, Bellinzona, Switzerland
6. Clinical School, University of New South Wales, Sydney, 2052, Australia.

**Keywords:** acute kidney injury, acute kidney disease, artificial intelligence, machine learning, prediction, real-world electronic health records

**Abstract word count:** 300

**Text word count:** 3922

**Reference number:** 23

**Table number:** 4

**Figure number:** 6

**Supplemental material (14 items)**

**Corresponding author**: Antonio Bellasi, MD, PhD. Service of Nephrology, Ospedale Regionale di Lugano, Ospedale Civico, Ente Ospedaliero Cantonale, Via Tesserete 46, 6903, Lugano, Switzerland. Email: [antonio.bellasi@eoc.ch](mailto:antonio.bellasi@eoc.ch); Università della Svizzera Italiana (USI), Lugano, Switzerland. Email: [antonio.bellasi@usi.ch](mailto:antonio.bellasi@usi.ch)

**Supplemental material 14 items**

1. **EOC data and setting**
2. **ML model to predict AKI occurrence during the first 24 hours of hospitalization**
3. **Supplemental Table 1:** diagnostic criteria for AKI
4. **Supplemental Table 2**: demographic, administrative, and laboratory data of the subjects experiencing AKI during the first day of hospitalization
5. **Supplemental Figure 1:** Renal function trajectories during hospitalization. Based on renal function trajectories, three different patient groups are identified: A) patients with normal renal function during the entire hospitalization (NAKI); B) patients with AKI during hospitalization and renal function recovery within seven days from the establishment of the diagnosis of AKI (AKI); C) patients with AKI during hospitalization without renal function recovery within seven days from the establishment of the diagnosis of AKI (AKD).
6. **Supplemental Figure 2**: graphical representation of the machine learning (ML) data analysis pipeline
7. **Supplemental Figure 3:** occurrence of AKI during hospitalization. Most renal events (about 77%) of AKI episodes occur within the first three days of hospitalization.
8. **Supplemental Figure 4.** Time to last creatinine assessment and discharge home or elapsing of the seventh day from AKI
9. **Supplemental Figure 5:** length of stay according to renal function impairment
10. **Supplemental Figure 6:** The SHAP analysis of the ML model to predict AKI occurrence during the first 24 hours of hospitalization
11. **Supplemental Figure 7:** SHAP analyses of the ML model running at day 1 and predicting AKI to AKD transition (AKD_T1).
12. **Supplemental Figure 8:** SHAP analyses of the ML model to predict AKD transition at day 2 (AKD_T2).
13. **Supplemental Figure 9:** SHAP analyses of the ML model to predict AKD transition at day 3 (AKD_T3).
14. **Supplemental Figure 10:** SHAP analyses of the ML model to predict AKD transition at day 4 (AKD_T4).

**EOC data and setting**

The EOC-ICT Department centrally manages the hospital information system and its local information communication and technology (ICT) infrastructure. The EOC-ICT has developed several in-house clinical applications, such as the clinical notes manager, the admission-discharge-transfer system, the prescriptions manager (drugs, nurse, chemotherapy), the emergency room manager, and part of the laboratory information system. This allowed us to integrate and transform data to develop a single data warehouse covering all the hospital domains (clinic, administration, logistics, security, etc.) connected through shared IDs (e.g., patient and hospitalization IDs are the same across all the domains). The data warehouse serves as the foundational infrastructure for hospital management, clinical research, and the deployment of data science applications.

For this study, we extracted clinical and administrative data (such as patient age and gender, admission date and time, specialty admission ward, elective or non-elective status) and laboratory tests from the data warehouse. Laboratory tests included pre-hospitalization data (considering data up to one year before admission) and data generated during the hospitalization. Laboratory tests included creatinine, potassium, hemoglobin, neutrophils, lymphocytes, eosinophils, basophils cell counts, mean corpuscular volume, and mean corpuscular hemoglobin of red blood cells. The laboratory data underwent coding utilizing an internal standard convertible into international laboratory coding systems, such as the LOINC.

**ML model to predict AKI occurrence during the first 24 hours of hospitalization**

When the ML algorithm was used on the external dataset to predict AKI during the first 24 hours of hospital stay, we observed a similar diagnostic accuracy (AUC-ROC 0.83 - AUC-PRC 0.17) than what was observed for the ML algorithm used to predict AKI during the whole hospitalization. The algorithm had high sensitivity and negative predictive value (NPV 0.99), corroborating the potential of this approach to rule out subjects at low risk of developing AKI.

|  | AKI during the first 24 h |
| --- | --- |
| Hospitalizations | 34,579 |
| Cases | 1,362 |
| AKI prevalence | 4% |
| Metric | |
|  |  |
| AUC-ROC | 0.82 |
| AUC-PRC | 0.18 |
| PPV | 0.18 |
| NPV | 0.99 |

**Table legend**: AKI: acute Kidney Injury, ROC: Receiving Operator Curve, PRC: Precision-Recall Curve, AUC: Area Under the Curve, PPV: Positive Predictive Value, NPV: Negative Predictive Value.

**Supplemental Table 1:** diagnostic criteria for AKI

| **Rules in case of baseline serum creatinine available** | |
| --- | --- |
| AKI stage 1 | sCr decrease >=26.5 µmol/L in 2 days |
| AKI stage 1 | sCr increase from 1.5 to 1.9 times the baseline |
| AKI stage 1 | sCr increase >=26.5 µmol/L in 2 days |
| AKI stage 2 | sCr increase from 2 to 2.9 times the baseline |
| AKI stage 3 | sCr increase >= 3 times the baseline |
| AKI stage 3 | sCr decrease >=353.6 µmol/L in 2 days |
| AKI stage 3 | sCr increase >=353.6 µmol/L in 2 days |
| **Rules in case of baseline serum creatinine not available** | |
| AKI stage 1 | sCr decrease >=26.5 µmol/L in 2 days |
| AKI stage 3 | sCr decrease >=353.6 µmol/L in 2 days |
| AKI stage 3 | sCr increase >=353.6 µmol/L in 2 days |

**Supplemental Table 2**: demographic, administrative, and laboratory data of the subjects experiencing AKI during the first day of hospitalization

|  | ALL | AKI_NAKD | AKI_AKD |
| --- | --- | --- | --- |
|  | (N=1362) | (N=1096) | (N=266) |
| Age, Mean ± SD | 72.73 ± 15.25 | 72.77 ± 14.77 | 72.58 ± 17.10 |
| Missing | 0 (0%) | 0 (0%) | 0 (0%) |
| Female sex, % | 43% | 43% | 41% |
| Missing | 0 (0%) | 0 (0%) | 0 (0%) |
| Medical Speciality Medicine, % | 59% | 58% | 62% |
| Missing | 0 (0%) | 0 (0%) | 0 (0%) |
| Medical Speciality Urology, % | 9% | 10% | 5% |
| Missing | 0 (0%) | 0 (0%) | 0 (0%) |
| Urgency admission, % | 90% | 91% | 86% |
| Missing | 0 (0%) | 0 (0%) | 0 (0%) |
| Elective admission, % | 9% | 8% | 12% |
| Missing | 0 (0%) | 0 (0%) | 0 (0%) |
| Institute Emergency Medicine, % | 19% | 20% | 12% |
| Missing | 0 (0%) | 0 (0%) | 0 (0%) |
| Institute Surgery, % | 23% | 24% | 19% |
| Missing | 0 (0%) | 0 (0%) | 0 (0%) |
| Creatinina baseline, Mean ± SD | 85.00 [66.00–112.00] | 88.00 [69.00–113.00] | 78.50 [58.00–102.00] |
| Missing | 297 (22%) | 283 (26%) | 14 (5%) |
| Creatinina 1st, Mean ± SD | 150.00 [110.00–212.00] | 149.00 [110.00–204.00] | 160.00 [109.00–249.75] |
| Missing | 0 (0%) | 0 (0%) | 0 (0%) |

Table legend: data are presented as mean(standard deviation) or median[interquartile range] when appropriate. sCR: serum creatinine at baseline; sCr 1^st^: first serum creatinine available during hospitalization

**Supplemental Figure 1:** Renal function trajectories during hospitalization. Based on renal function trajectories, three different patient groups are identified: A) patients with normal renal function during the entire hospitalization (NAKI); B) patients with AKI during hospitalization and renal function recovery within seven days from the establishment of the diagnosis of AKI (AKI); C) patients with AKI during hospitalization without renal function recovery within seven days from the establishment of the diagnosis of AKI (AKD).

**
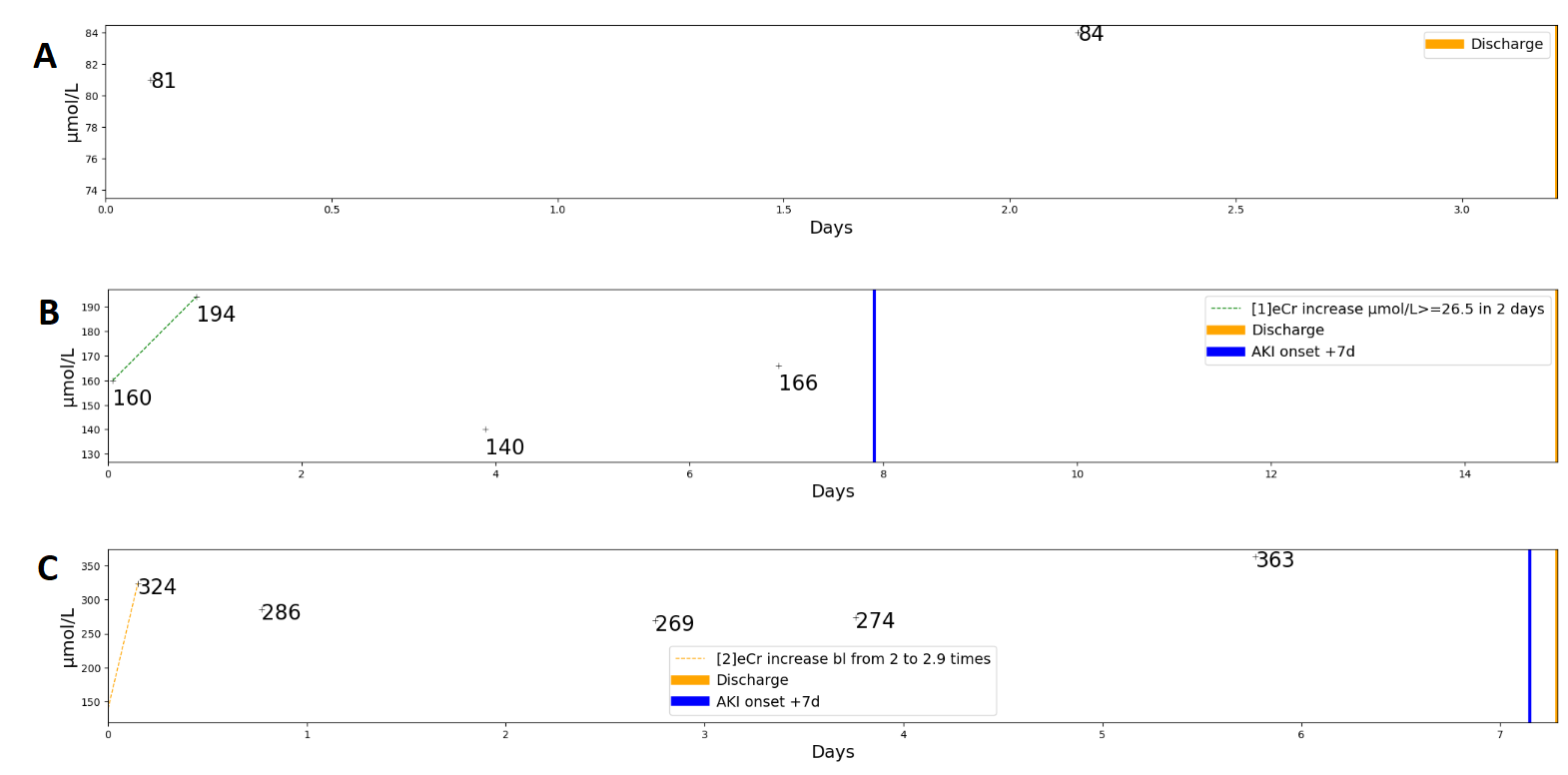
**

Figure legend:

A) NAKI trajectory: no renal function impairment

B) AKI trajectory: renal function impairment and recovery within 7 days

C) AKD trajectory: renal function impairment and no recovery within 7 days

**Supplemental Figure 2**: **graphical representation of the machine learning (ML) data analysis pipeline.** The dataset was divided into a training and validation set (90%) and an external validation set (10%). The chronological order of cases was maintained, with newer cases assigned to the external validation set to better mimic real-world conditions. The training and validation set was utilized to evaluate various hyperparameters of the LightGBM (LGB) model through k-fold cross-validation (3 repetitions), randomly generating hyperparameter configurations. The best-performing configuration, identified as having the highest mean AUC-PRC, was chosen as the best model. This model was trained on the entire training and validation set and calibrated to refine its predicted probabilities, ensuring they accurately reflect the actual likelihood of outcomes. The calibrated model, referred to as the ML model, was then assessed for its performance on the external validation set

**
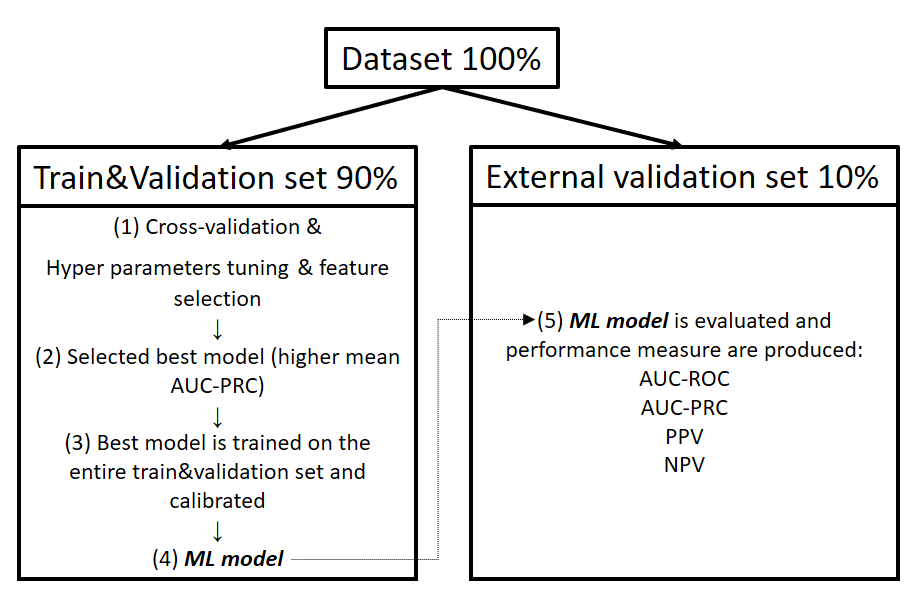
**

**Supplemental Figure 3:** occurrence of AKI during hospitalization. Most renal events (about 77%) of AKI episodes occur within the first three days of hospitalization. Specifically, 41% occur within 0-24 hours, 28% between 25-48 hours, 8% between 49-72 hours, and 23% after 72 hours.


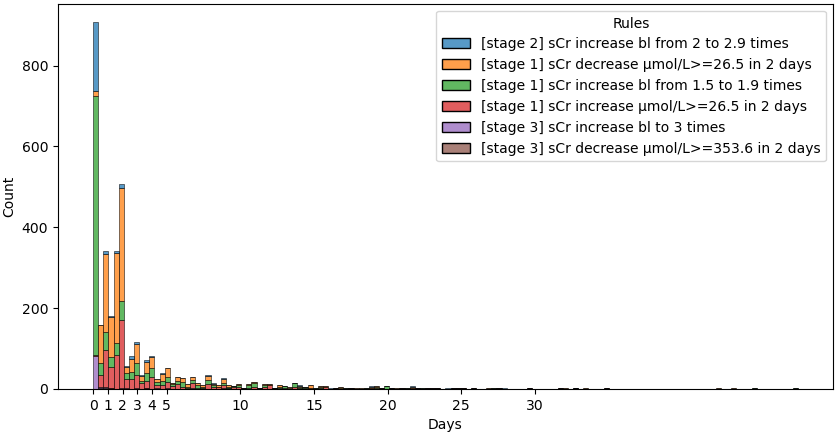


**Supplemental Figure 4.** Time to last creatinine assessment and elapsing of the seventh day from AKI. (A) The boxplot describes the distribution of the number of days missing to complete the follow-up of 7 days from AKI onset, as mandated by the definition of AKD. The median number of days is 3,9. In case of a followup shorter than 7 days, the last value of creatinine available was used to define the renal function trajectory. In the 2 cartoons at the bottom, there are 2 examples of follow-up shorter than 7 days. In example 1 (B), the last sCr value is available 3.26 days before the seventh day from AKI. In example 2 (C), the last sCr is available 0.53 days before the seventh day from AKI. Notably, among the 266 AKD cases, 139 were discharged before the seventh day elapsed from the onset of AKI (as seen in example 1).

**
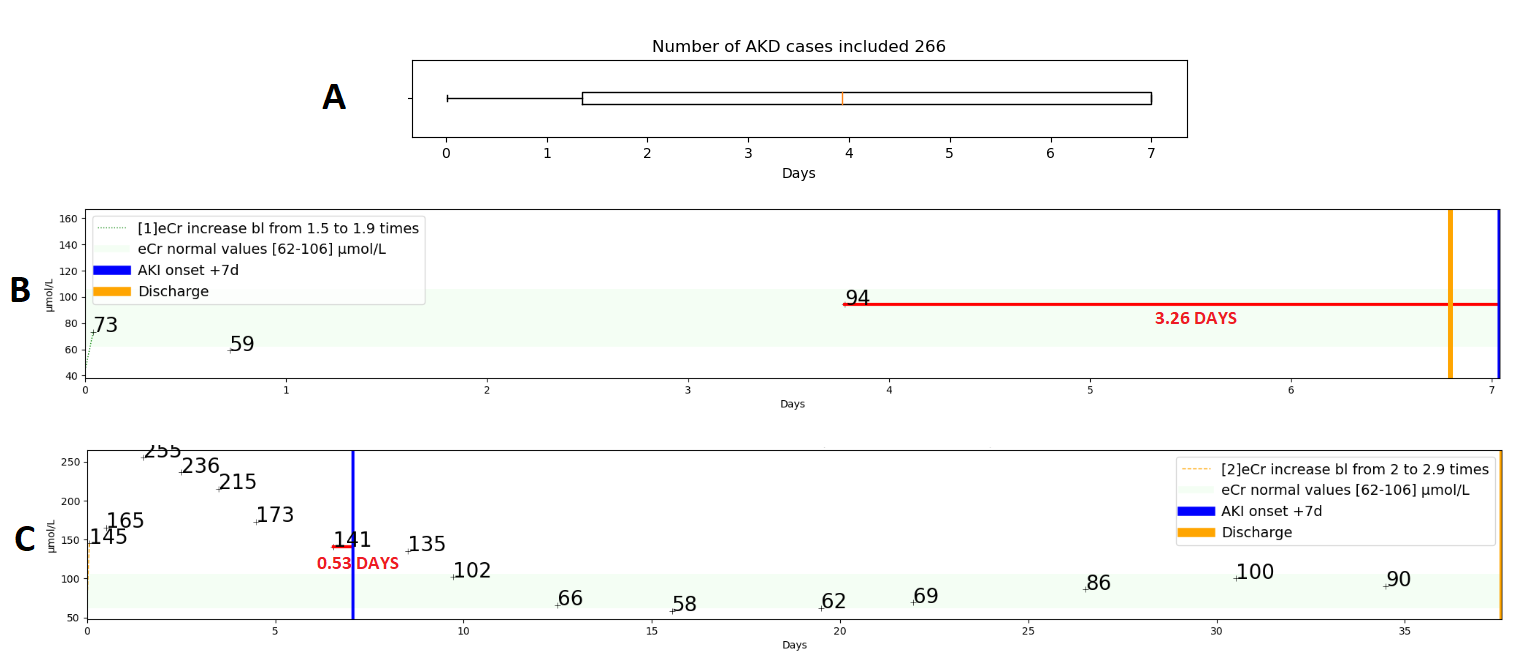
**

**Supplemental Figure 5:** length of stay according to renal function impairment

**
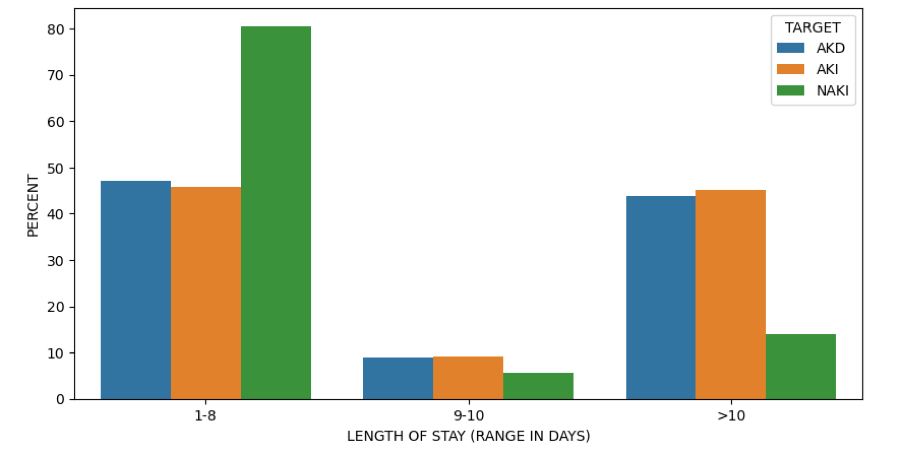
**

**Supplemental Figure 6:** The SHAP analysis reveals that the type of hospital admission (elective vs. urgent hospital admission) is the most important feature in predicting renal function impairment.


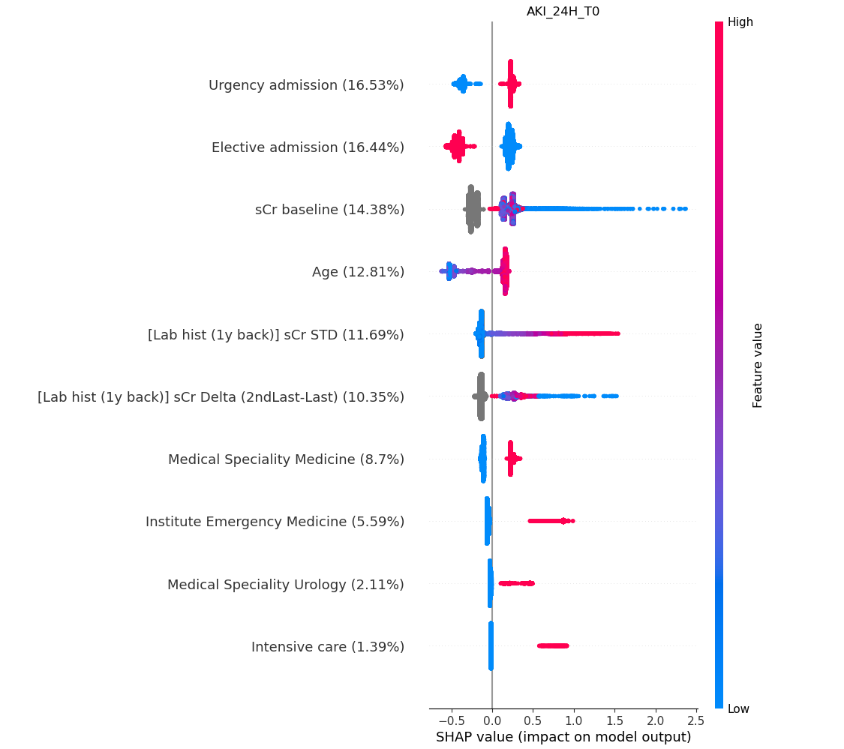


**Figure legend**: sCr: serum creatinine; ALATGPT NTH: alanine aminotransferase; STD: Standard deviation.

Between the square brackets, the time point considered is indicated. For example, [lab hist (1y back)]sCr maximum refers to the maximum serum creatinine value in the 365 to 7 days before hospitalization.

Delta signifies a change that occurs during hospitalization. The time points used to calculate the delta are indicated in round brackets. For example, [lab hist (1y back)] sCr Delta (2ndlast-last) refers to the change in serum creatinine (micromol/l) between the last 2 values available in the 365 to 7 days before hospitalization.

**Supplemental Figure 7:** SHAP analyses of the ML model running at day 1 and predicting AKI to AKD transition (AKD_T1).


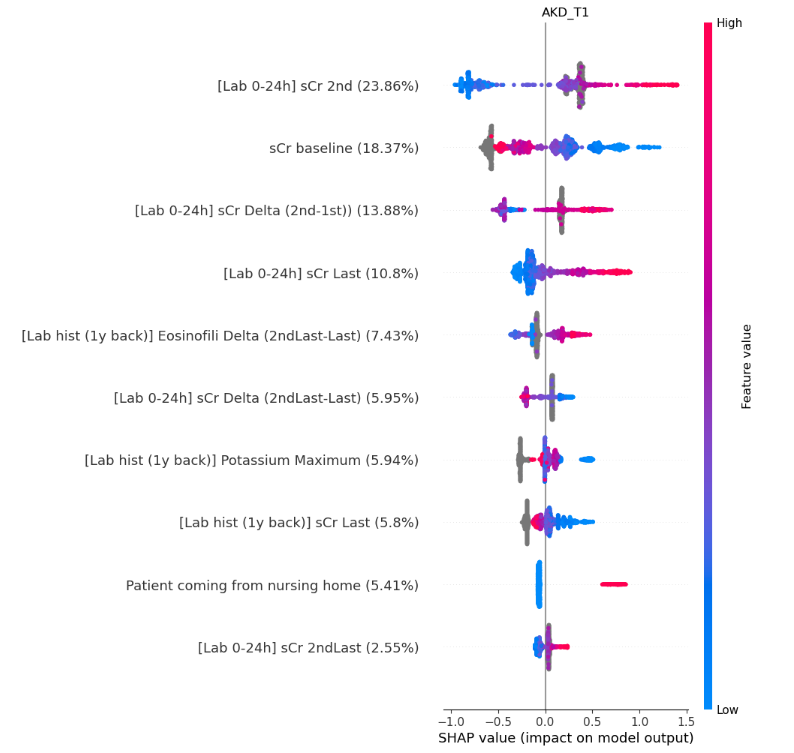


**Figure legend**: sCr: serum creatinine; ALATGPT NTH: alanine aminotransferase; STD: Standard deviation.

Between the square brackets, the time point considered is indicated. For example, [lab hist (1y back)]sCr maximum refers to the maximum serum creatinine value in the 365 to 7 days before hospitalization.

Delta signifies a change that occurs during hospitalization. The time points used to calculate the delta are indicated in round brackets. For example, [lab 0-24] sCr Delta (2nd-1st) refers to the change in serum creatinine (micromol/l) between day 2 and day 1.

**Supplemental Figure 8:** SHAP analyses of the ML model to predict AKI to AKD transition at day 2 (AKD_T2).


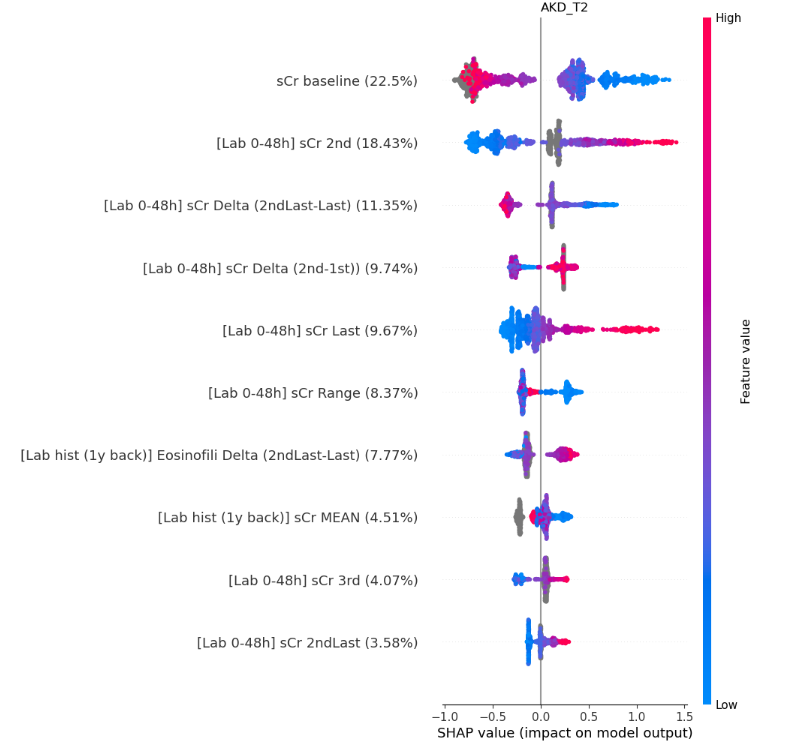


**Figure legend**: sCr: serum creatinine; ALATGPT NTH: alanine aminotransferase; STD: Standard deviation.

Between the square brackets, the time point considered is indicated. For example, [lab hist (1y back)]sCr maximum refers to the maximum serum creatinine value in the 365 to 7 days before hospitalization.

Delta signifies a change that occurs during hospitalization. The time points used to calculate the delta are indicated in round brackets. For example, [lab 0-24] sCr Delta (2nd-1st) refers to the change in serum creatinine (micromol/l) between day 2 and day 1.

**Supplemental Figure 9:** SHAP analyses of the ML model to predict AKI to AKD transition at day 3 (AKD_T3).


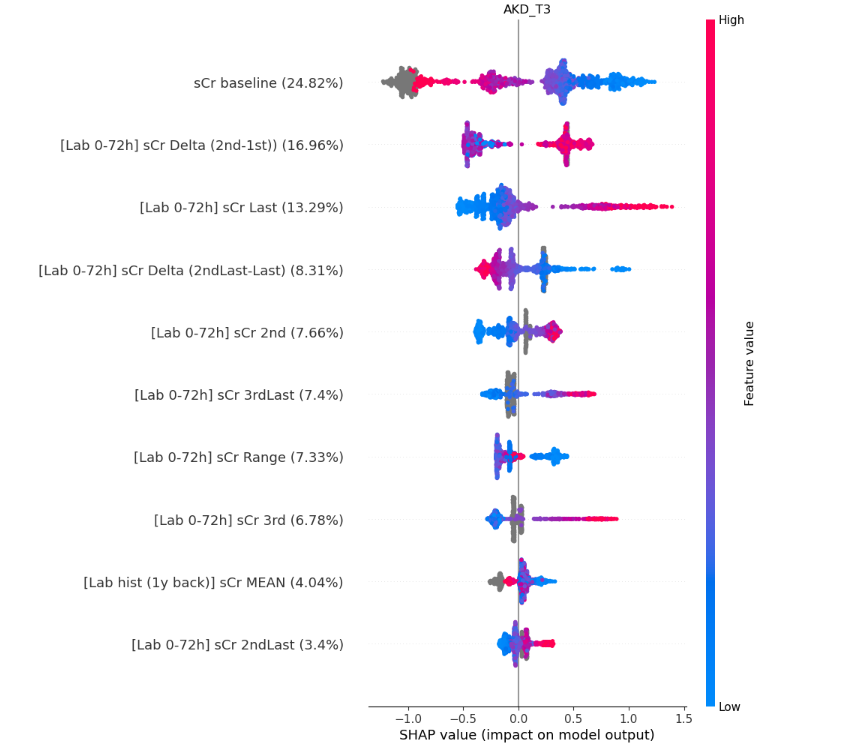


**Figure legend**: sCr: serum creatinine; ALATGPT NTH: alanine aminotransferase; STD: Standard deviation.

Between the square brackets, the time point considered is indicated. For example, [lab hist (1y back)]sCr maximum refers to the maximum serum creatinine value in the 365 to 7 days before hospitalization.

Delta signifies a change that occurs during hospitalization. The time points used to calculate the delta are indicated in round brackets. For example, [lab 0-24] sCr Delta (2nd-1st) refers to the change in serum creatinine (micromol/l) between day 2 and day 1.

**Supplemental Figure 10:** SHAP analyses of the ML model to predict AKI to AKD transition at day 4 (AKD_T4).


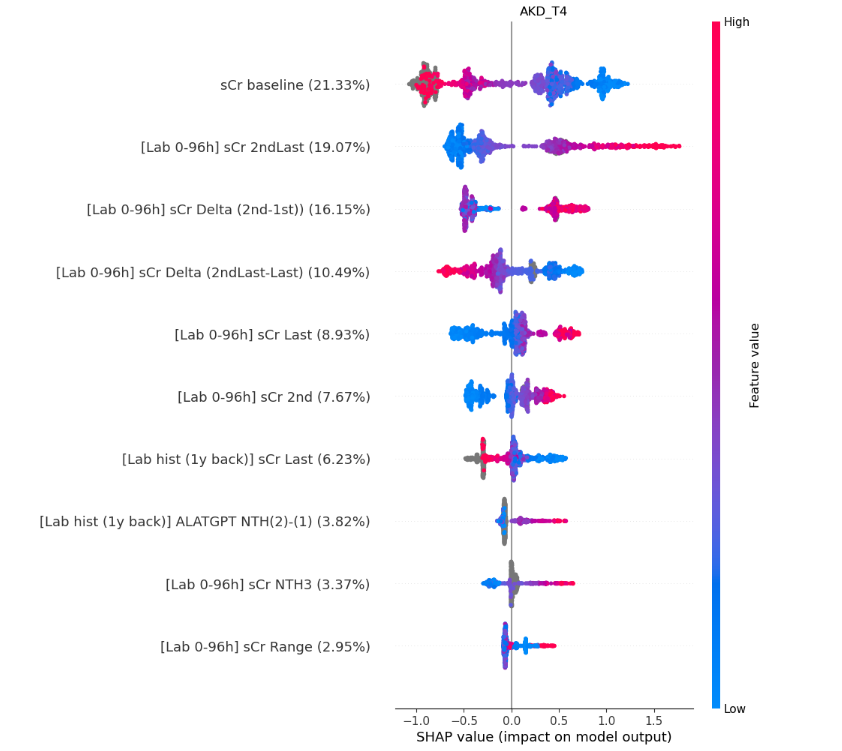


**Figure legend**: sCr: serum creatinine; ALATGPT NTH: alanine aminotransferase; STD: Standard deviation.

Between the square brackets, the time point considered is indicated. For example, [lab hist (1y back)]sCr maximum refers to the maximum serum creatinine value in the 365 to 7 days before hospitalization.

Delta signifies a change that occurs during hospitalization. The time points used to calculate the delta are indicated in round brackets. For example, [lab 0-24] sCr Delta (2nd-1st) refers to the change in serum creatinine (micromol/l) between day 2 and day 1.
