# Supplementary material for: Usability of machine learning algorithms based on electronic health records for the prediction of acute kidney injury and transition to acute kidney disease: A proof of concept study
Source: PLoS One. 2025 Jul 1;20(7):e0326124. doi: 10.1371/journal.pone.0326124 (PMC12212546; doi:10.1371/journal.pone.0326124)
Supplement: Attachment — Submitted filename: 2025_02_04_rebuttal.docx [file pone.0326124.s003.docx]

Emily Chenette,

Editor-in-Chief,

PlosOne.

February 7^th^, 2025

Subject: Rebuttal (Manuscript Reference Number PONE-D-24-42164)

Dear Professor Emily Chenette,

Enclosed is a revised manuscript by Ruinelli et al. entitled: “Usability of Machine Learning Algorithms Based on Electronic Health Records for the Prediction of Acute Kidney Injury and Transition to Acute Kidney Disease: A Proof of Concept Study."

We thank you and the reviewers for the time spent reviewing the paper. We would like to resubmit a corrected version of the manuscript that incorporates the comments and observations.

While the original meaning of the manuscript remains unchanged, the new version (attached is a copy showing deletions highlighted in blue and strikethrough font, additions in red font, and a clean version) has been refined. Also, please note that some of the pictures were edited due to the data analysis pipeline refinement suggested by reviewer #3 (highlighted in the text).

Below, you will find a point-by-point response to the reviewers' comments.

We are open to any additional comments or questions you may have.

Sincerely,

Antonio Bellasi,

On behalf of all co-authors

**Point-by-point answers to the reviewers**

**Reviewer #1:**

Hello! I just reviewed the article entitled "Usability of Machine Learning Algorithms based on Electronic Health Records for the Prediction of Acute Kidney Injury and Transition to Acute Kidney Disease: a Proof of Concept Study" by Lorenzo Ruinelli et al. With this study, the authors proved that ML algorithms could predict AKI and/or AI to AKD transition.

Overall, the study is well-written, in good English, with a good flow, and logical.

**Authors:** We appreciate the reviewer's time and effort in reviewing the paper and are grateful for the praise.

Before considering publishing the article, I have some suggestions for the authors, as minor revisions:

1. Please revise the continuous variables regarding the normal distribution. I saw some high SD compared to the means, and this suggests that the data is not normally distributed.

Authors: We thank the reviewer for raising this point. We believe it pertains to the figures reported in Table 2 and Supplemental Table 2. We have carefully revised it in the latest version of the manuscript. Please note that to enhance readability, we have also added a table legend (highlited in red fonts).

2. In table 2 and supplemental table 2, you present the estimated GFR 1st. I assume this is the calculated GFR from day 1 of hospital admission. Due to the fact that most of the patients presented already with AKI in the first day of hospital admission, I consider the use of eGFR to be inappropriate. The use of eGFR in context of acute kidney injury is unreliable. Nevertheless, you could replace the eGFR from day one with baseline eGFR - calculated with from the baseline creatinine. In this case, I would expect that patients who develop AKD to present lower baseline eGFR.
Authors: We agree with the remark. To avoid misinterpretation and considering that this information is not available for all patients, we decided to remove eGFR from Table 2 and Supplemental Table 2.

3. An important factor for transition to AKD from AKI would be the AKI cause. I consider that you should add in the limitations paragraph the absence of AKI cause in your model. Intrinsic AKI is proved to increase the risk of AKI prolongation towards AKD. Given this aspect, you indeed presented in the discussion section the clinical importance of AKI and/or AKI to AKD progression. I consider you could expand this paragraph and discuss the clinical solutions stratified by AKI causes (prerenal, intrinsic and postrenal - as many of AKI were from the urology department). Nevertheless, you should discuss the importance of the nephrotoxic medication avoidance in context of AKI.

**Authors:** We agree with the reviewer that the cause of AKI and AKD would likely improve the model's accuracy. However, this piece of information is not available in our dataset. We have expanded the discussion session to highlight this limitation. We added this sentence: “First, this retrospective study utilizes data from a single healthcare system, and information on the etiology of renal failure is missing. While this last piece of information would likely have enhanced the model's accuracy, our sequential approach, utilizing real-world data, demonstrates its potential in predicting the transition from AKI to AKD while overcoming this inherent data limitation.” (Page 11)

4. Overall, a good article, logical and great flow. In the end, I would like to add that besides the diagnosis and prediction of AKI/AKD occurrence, the recognition of AKI is of great importance. As you clearly stated in your article, the AKI diagnosis rate was reduced and the nephrology reference was even lower. 5. Good luck!

**Authors:** We appreciate the reviewer’s comment and kind words.

**Reviewer #2:**

Dear Authors, I have read your work with great interest. It presents a highly intriguing application of AI in a very active research area. However, I would like to offer some observations and requests to enhance the paper’s clarity, which might be challenging to follow, and to highlight aspects that I believe could be valuable for the readers:

**Authors:** We thank the reviewer for the time spent reviewing the paper and for the comments made.

Incidence of AKI: The AKI incidence reported in your study appears to be lower than what is typically documented in the literature, which estimates it to be around 20% in hospitalized patients (DOI: 10.1016/S0140-6736(16)30240-9). Do you have any opinions or comments on this discrepancy?

**Authors:** We believe that the difference in AKI incidence is influenced by the case mix and the setting considered. For instance, Mehta and colleagues, in the article referenced by the reviewer, report on a multinational cross-sectional study evaluating AKI incidence in both hospital and non-hospital settings, as well as in high-income and low-income countries. All of these factors have been linked to the risk of renal failure. To better elucidate this point we added a sentence in the discussion: “*Although significant variability in the incidence of AKI is reported depending on the settings and case-mix,* …” (see page 10)

**Definition of AKI.** The AKI definition used in your study is unclear. According to KDIGO guidelines, AKI diagnosis requires a creatinine increase within 48 hours or over 7 days from a baseline value. In your study, however, the baseline value could date back up to one year. Additionally, when no baseline value was available, you diagnosed AKI based on a two-day creatinine increase, but what was this increase compared against?

**Authors:** We appreciate the reviewer's comments. To ensure comparability, we applied the extended KDIGO guidelines for defining AKI. Like other authors, we adopted this approach because it has been shown that this definition of AKI detects cases occurring earlier in hospitalization, which is similarly associated with worse outcomes (we refer to the methods section, page 5 of the manuscript). We reported the criteria for defining AKI during hospitalization in Supplemental Table 1. Depending on the availability of baseline creatinine, a different set of rules is used.

There is no consensus on the definition of baseline creatinine. However, according to the research by *Niemantsverdriet et al. BMC Nephrol 2021; 22:371*, a similar effect on the diagnosis of AKI can be expected from the lowest sCr value during the time frame of 7 to 365 days before hospitalization. We adopted this definition to encompass as many subjects with baseline serum creatinine (sCr) as possible. In our view, the limited availability of serum creatinine is a major limitation of the existing literature on this subject.

To better explain this point, we have added a sentence in the methods section to clarify it further (page 5).

**Definition of Renal Recovery** How was renal recovery defined in your study?

**Authors:** We thank the reviewer for the remark and the lack of clarity in the text. Renal recovery is defined by the absence of AKI to AKD transition. To better clarify this aspect, we added a sentence in the methods: “Renal recovery was defined as a reduction in sCr within 7 days from AKI to a value less than 1.5 times the baseline value or a sCr value lower than 353.6 μmol/L if the baseline sCr is not available”. (page 6)

**AKI Analysis**: It is important to emphasize that AKI occurring during the first days of hospitalization differs from that developing later. Notably, there is a well-documented distinction between community-acquired AKI and hospital-acquired AKI, which may have different causes and outcomes (DOI: 10.1053/j.ajkd.2023.10.009, DOI: 10.2215/CJN.07920713). For example, a recent prospective study demonstrated that the primary risk factor for community-acquired AKI is the use of nephrotoxic drugs (DOI: 10.1016/j.ejim.2024.09.016). However, this study lacks a detailed analysis of AKI causes and does not consider this factor.

**Authors:** We agree with the reviewer that the cause of AKI and AKD would likely improve the model's accuracy. However, this piece of information is not available in our dataset. We have expanded the discussion session to highlight this limitation. We added this sentence: “First, this retrospective study utilizes data from a single healthcare system, and information on the etiology of renal failure is missing. While this last piece of information would likely have enhanced the model's accuracy, our sequential approach, utilizing real-world data, demonstrates its potential in predicting the transition from AKI to AKD while overcoming this inherent data limitation” (Page 11)

Another observation is related to the models used to assess AKD risk in this study. These models are based on data collected during the early days of hospitalization, excluding AKI cases that develop later—cases that represent true hospital-acquired AKI and are generally associated with worse prognoses. I suggest that the authors discuss these aspects in more detail (some of which are already partially addressed) and include them as study limitations.

**Authors:** We agree with the reviewer’s remark. However, this is a proof-of-principle study to evaluate the feasibility of a real-time data analysis approach to improve AKI and AKD prediction. In light of the overall mean hospitalization length, which is shorter than 7 days, as required by the KDIGO definition of AKD, we decided to focus on those subjects experiencing AKI during the first 24 hours of their hospital stay. In case of death, patient discharge, or lack of renal function assessment seven days after AKI, AKD was defined according to the last sCr value available (right censoring). While this approach would enhance sensitivity, it would also increase the algorithm's negative predictive power, a desirable feature for a diagnostic tool designed to rule out patients with low or no risk of developing renal function impairment. We have added these details to the methods section (page 6)

**Comparison of AKI and AKD** **Patients** A comparison between AKI patients who recover and those who progress to AKD would be interesting. Currently, Supplementary Table 2 lacks statistical analysis for this comparison.

**Authors:** We agree with the reviewer that a comparative analysis between AKI and AKD patients would be of interest. However, this is outside the scope of our current research, which aims to develop an algorithm to predict the occurrence of AKI during hospital stays and the transition from AKI to AKD, using data collected longitudinally in the initial days of hospitalization and before the onset of AKD. The clinical relevance of this approach hinges on the possibility (if validated in an external cohort) of personalized care and potentially identifies patients who require a nephrological referral. In fact, only 4% and 8% of hospitalizations complicated by AKI and AKD, respectively, are referred to a nephrologist in EOC. These data are presented in the results section on page 8.

(1)

**SHAP Analysis Variables**: The SHAP analyses include variables that are not explained, such as sCr MAX/SD/LAST. What do these variables represent, and how do they differ from sCr baseline? Additionally, how are the deltas calculated? Providing explanations for these factors would enhance the paper’s comprehensiveness.

**Authors:** We apologize for the lack of clarity. We have now added figure legends in Figure 3B, Figure 6, Supplemental figures 6A and Supplemental Figures 6B to 6D. Please note that the pictures were edited due to the data analysis pipeline refinement suggested by reviewer #3.

**Discrepancy Between Administrative and Biochemical Data**: A recent study highlighted the discrepancy between administrative and biochemical data in AKI diagnosis and showed the existence of different patterns of AKI recognition in hospitals (DOI: 10.1093/ckj/sfae231). Including a discussion on this point could be useful, particularly for guiding future research.

**Authors:** We completely agree with the reviewer that AKI reporting is highly heterogeneous, and studies based on administrative or laboratory data frequently report varying prevalence rates of AKI. We have added the reference and the sentence to the manuscript. (page 3, page 11) and a new reference (reference #11)

**Supplementary Figure 1** is unclear and could benefit from further clarification.

**Authors:** We thank the reviewer for the comment. We noticed that we inadvertently switched figures B and C in Supplemental Figure 1. We have now corrected the two figures. We apologize for the inconvenience. Furthermore, please note that as per the reviewer #3 suggestions, we have added a new supplemental figure. Hence the number of the supplemental figures has been updated. (page 34).

**Reviewer #3**:

The study concerns a topic not treated in deep, which is of interest in a large number of patients admitted out of Intensive Care Units.

**Authors:** We appreciate the reviewer's time and effort in reviewing the paper.

I have some key questions on the methods and results of this study.

The process of evaluation of machine learning algorithms is lacking of two steps: the first is the calibration and the second is the external validation. The calibration test which is useful to calibrate the predictive model. The external test set, which is necessary to prove the model performance and its ability to generalize on different geographical populations. Consequently I suggest to review in depth the process of ML algorithms generation, because we don’t know if the model is well calibrated and if the algorithm can be widely applied.

**Authors:** We appreciate the reviewer’s feedback regarding the lack of clarity on this point. The dataset was divided into a training and validation set (90%) and an external validation set (10%). The chronological order of cases was maintained, with newer cases assigned to the external validation set to better mimic real-world conditions. The training and validation set was utilized to evaluate various hyperparameters of the LightGBM (LGB) model through k-fold cross-validation (3 repetitions), randomly generating hyperparameter configurations. The best-performing configuration, identified as having the highest mean AUC-PRC, was chosen as the best model. This model was trained on the entire training and validation set and calibrated to refine its predicted probabilities, ensuring they accurately reflect the actual likelihood of outcomes. The calibrated model, referred to as the ML model, was then assessed for its performance on the external validation set. Supplemental Figure 2 provides a graphical representation of the revised ML pipeline (page 33).

Furthermore, we reported these limitations in the discussion and changed the clusion as followoing: “*However, without appropriate external validation, caution must be exercised in interpreting the current results. Future efforts and specific initiatives are required to ascertain whether the implementation of an AI decision-support tool can enhance patient care, improve outcomes, and optimize healthcare resource allocation”* (page 13).

Other aspects:

1) AKI and AKD occurrence and association with outcome. The authors report 3305 subjects who developed AKI during hospital stay. Then they report AKI in the first 24h = 1362 pts, in the 48 h 2279 and in the 72 hrs 2556. I think that it should be better to describe AKI periods: 0-24hrs, 25-48 hrs, 49-72 hrs, > 72hrs. This information can be added to supplemental figure 2.

**Authors:** We appreciate the reviewer’s feedback and have incorporated this valuable information into the caption of Figure 2 (page 16), supplemental Figure 3 (please note the supplemental figures number has been edited) (page 35)

2) A better explanation is needed for AKD transition. In this case it is not clear the definition of AKD because only 47.8% of patients with AKI in the first 24 hrs completed 7 days of observation needed for AKD assessment (see definition of AKD reported on methods sections).

**Authors:** We agree with the reviewer’s remark. However, this is a proof-of-principle study to evaluate the feasibility of a real-time data analysis approach to improve AKI and AKD prediction. In light of the overall mean hospitalization length, which is shorter than 7 days, as required by the KDIGO definition of AKD, we decided to focus on those subjects experiencing AKI during the first 24 hours of their hospital stay. In case of death, patient discharge, or lack of renal function assessment seven days after AKI, AKD was defined according to the last sCr value available (right censoring). While this approach would enhance sensitivity, it would also increase the algorithm's negative predictive power, a desirable feature for a diagnostic tool designed to rule out patients with low or no risk of developing renal function impairment. We have added these details to the methods section (page 6)

3) Positive predictive value is reported, but the percentage is low. A comment is needed. Could we consider useful a prediction model with such as margin of error? In discussion this aspect is not reported or commented.

**Authors:** We believe that the progressively higher sensitivity and negative predictive power (NPV from 94% on day 2 to 98% on day 4) suggest the potential of the algorithm to rule out subjects at low risk of developing renal function impairment.

We have added these details to the methods and in the discussion (page 6, page 11 and page 12)

**Reviewer #4**:

The study is very articulate and analyzed an interesting role for ML in predicting AKI or AKD occurrence in acute hospitalized patients. Nevertheless, some methodological limits (e.g. retrospective, mono-centric and selection criteria) I think this study may add interesting insights for ML in clinical practice. Below, the Authors can find my comments about the manuscript.

Minor revisions

A) Abstract:

- "AKI and AKD" are used appropriately, but the definition of these conditions could be included, considering that the abstract is addressed to an audience that may not be familiar with these conditions. (e.g. According to the KDIGO guidelines?). Is it due to the word-limit?

**Authors**: Although we agree with the reviewer, the 300-word constraint unfortunately prevents us from adding what the reviewer suggests. However, a definition of AKI and AKD is provided at the beginning of the abstract.

- I suggest reformulating as follow: “…The majority of AKI episodes (77%) occurred within the first three days of hospitalization”.

**Authors:** the text has been revised according to the reviewer's suggestions (page 2).

- “AKI and AKD complicated 10% and 1.5% of hospitalizations”: respectively?

**Authors:** the text has been revised according to the reviewer's suggestions (page 2).

B) Aims

-Better “The aims of the study are as follows:” and “to minimize right censoring due to patient mortality or discharge before the completion of 7 days of observation following the onset of AKI;”.

**Authors:** the text has been revised according to the reviewer's suggestions (page 5).

Major revisions Some comments for the Authors, to which I would appreciate their responses.

1) The main limitation of the Study may be the reduced representativeness of the enrolled patient population, as it excludes groups with significant renal comorbidities. However, if the aim is to develop a highly specific model for predicting AKI in a low-risk population, this exclusion may be appropriate." Indeed, by excluding patients with severe pre-existing renal disease, the dataset likely includes a higher proportion of individuals with low-risk for AKI or AKD and potentially increasing the NPV. This may limit the ML model's generalizability, as excluding patients with severe renal impairment reduces dataset representativeness for populations with higher prevalence of CKD or significant comorbidities

**Authors:** We thank the reviewer for the comment. We have added it as a study limitation on page 12: “Fifth, caution should be exercised when evaluating the results of this study. The absence of proper external validation and the exclusion of subjects with pre-existing advanced renal function impairment likely biases our study cohort towards a low-risk population for AKI or AKD, potentially increasing the NPV and limiting the algorithm's generalizability. While the use of this approach to individuals with advanced CKD or significant comorbidities cannot be extrapolated…”

2) The exclusive use of sCr to define AKI and AKD, while widely accepted, may not fully include the complexity of AKI. sCr is influenced by factors such as age, sex, muscle mass, and hydration status, potentially distorting renal function assessment. Additionally, in patients with pre-existing conditions like dehydration or malnutrition, sCr may not accurately reflect renal function. For this reason, it would be interesting to have at least the reason for hospitalization and some information on comorbidities to better guide the clinical utility of the ML model.

**Authors:** We agree with the reviewer that the cause of AKI and AKD would likely improve the model's accuracy. However, this piece of information is not available in our dataset. We have expanded the discussion session to highlight this limitation. We added this sentence: “First, this retrospective study utilizes data from a single healthcare system, and information on the etiology of renal failure is missing. While this last piece of information would likely have enhanced the model's accuracy, our sequential approach, utilizing real-world data, demonstrates its potential in predicting the transition from AKI to AKD while overcoming this inherent data limitation.” (Page 11)

3) The extended definition of AKI, including serum creatinine increases >26.5 μmol/L or >1.5 times baseline, may lead to overdiagnosis by identifying minor, clinically non-significant renal function fluctuations as AKI. While it improves early detection, these minor changes often may lack prognostic relevance, potentially increasing sensitivity at the expense of specificity affecting the clinical power of ML. Please, Authors comment this aspect in the text.

**Authors:** We appreciate the reviewer's comments. To ensure comparability, we applied the extended KDIGO guidelines for defining AKI. Like other authors, we adopted this approach because it has been shown that this definition of AKI detects cases occurring earlier in hospitalization, which is similarly associated with worse outcomes (we refer to the methods section, page 5 of the manuscript).

4) The retrospective design depends on potentially incomplete EHR data, which may affect accuracy. This could be a limitation for generalization that needs to be better considered in the discussion.

**Authors:** We agree with the reviewer that the retrospective design of the study is a limitation (pages 11 and 12).

5) Right censoring due to death, discharge, or missing renal data within 7 days could skew results. The progressively smaller datasets for AKD models (T1-T4) may reduce ML model’s stability and generalizability. Additionally, time-specific models may fail to capture slower or atypical disease progressions, limiting their applicability.

**Authors:** We agree with the reviewer’s comment. However, this is a proof-of-principle study to evaluate the feasibility of a real-time data analysis approach to improve AKI and AKD prediction. In light of the overall mean hospitalization length, which is shorter than 7 days as required by the KDIGO definition of AKD, we decided to focus on those subjects experiencing AKI during the first 24 hours of their hospital stay. In the event of death, patient discharge, or a lack of renal function assessment seven days after AKI, AKD was defined according to the last sCr value available (right censoring). While this approach would enhance sensitivity, it would also increase the algorithm's negative predictive power, a desirable feature for a diagnostic tool designed to rule out patients with low or no risk of developing renal function impairment. We have added these details to the methods section (page 6) and highlighted in the study limitations that caution should be exercised in interpreting these results due to the lack of external validation and the potential drawbacks of the study (pages 11 and 12).

In details, other comments about Discussion and Conclusion that I underline to the Authors.

- Only 35% of AKI and 52% of AKD cases were recorded, with a notably low rate of nephrology referrals. Could this suggest a significant proportion of events clinically not impactful?

**Authors:** We respect the reviewer’s opinion, but we are inclined to think that the underdiagnosis of AKI and AKD is the reason for the undercoding and low nephrology referral rate of these cases.

- The median follow-up duration of 4.1 days, combined with right censoring due to discharge or death, may have led to an overestimation of the ML’s ability to predict long-term AKD outcomes. This requires further discussion.

**Authors:** Please refer to the answer for point 5. We have further discussed these aspects in the study limitations(pages 11 and 12). We remain available to comment on these aspects in the study discussion if the reviewer feels strongly about it.

- Serum creatinine (sCr) data were available in only 48% of cases. This limitation could significantly impact the ML model’s accuracy and should be addressed in the discussion.

**Authors:** the limited availability of serum creatinine is a significant limitation of the existing literature on this subject. There is no consensus on the definition of baseline creatinine, however, according to the research by *Niemantsverdriet et al. BMC Nephrol 2021; 22:371*, a similar effect on the diagnosis of AKI can be expected from the lowest sCr value from 7 to 365 days before hospitalization. We adopted this definition to encompass as many subjects with baseline serum creatinine (sCr) as possible.

To better explain this point, we have added a sentence in the methods section to clarify it further (page 5) and added reference #20 (page 14).

- The AUC-ROC values (0.76–0.88), alongside missing data on patient comorbidities and medication use, are critical limitations that could affect both the ML model's performance and its clinical applicability. These aspects deserve more comprehensive consideration.

**Authors:** We have further discussed these aspects in the study limitations (pages 11 and 12). We believe this is a *proof-of-concep*t study that deserves external validation before implementing the algorithms. However, we remain convinced that our preliminary results suppport the use of repeat application of ML during hospital stay to assist AKI and AKD rik profiling and that ML may be a useful tool to clinicians. We are available to address these aspects in the study discussion if the reviewer feels strongly about it.

- The study is based on retrospective data from a single healthcare system. This raises concerns about the generalizability of the findings, which should be better contextualized in the discussion.

**Authors:** the retrospective nature of the study from a single healthcare system have been added among the study limitations (pages 11 and 12).

- Finally, it may be valuable to explore the relationship between hospitalization length and the occurrence of AKI in more detail. That Said, could Supplemental Figure 3 be integrated into the main text to provide greater clarity and emphasis?

**Authors:** We appreciate the comment. However, we believe that this could mislead the interpretation of the AKD models. These models focus on those subjects with AKI during the first day of hospitalization and not on the entire cohort of subjects experiencing AKI throughout their hospitalization. Since we lack data on the etiology of AKI, we decided not to explore the relationship between AKI and the length of hospital stay, choosing instead to report these findings as descriptive only in the supplemental material.
