# Supplementary material for: Usability of machine learning algorithms based on electronic health records for the prediction of acute kidney injury and transition to acute kidney disease: A proof of concept study
Source: PLoS One. 2025 Jul 1;20(7):e0326124. doi: 10.1371/journal.pone.0326124 (PMC12212546; doi:10.1371/journal.pone.0326124)
Supplement: Attachment — Submitted filename: Response to Reviewers.docx [file pone.0326124.s004.docx]

**Point-by-point answers to the reviewers and Academic editor’s comments**

**Academic Editor’s comments:**

Please provide minor revision requested, in particular:

-Please try to describe a hypothetical sample size in order to indicate the statistical power of the ML results and their associated NPV, particularly given the low incidence of AKI and AKD.

**Answer:** We thank the editor for the comments and the opportunity to review the paper.

We appreciate the insightful comment regarding the necessity of describing a hypothetical sample size. As detailed in the answer to Reviewer #4, a *post hoc* calculation of the sample size in a retrospective study likely overestimates the study power. Instead, when Real-World Data (RWD) is utilized, it is important to ensure that the dataset is representative and sufficiently large to capture the variability of the outcome and predictor variables.

Regarding the low incidence of AKI in our cohort, we respectfully note that studies using established criteria typically report an AKI prevalence of 10-30% among unselected hospital admissions (see table). The wide range of reported AKI prevalence in admitted patients is likely due to various reasons, such as diagnostic criteria and administrative versus laboratory data used, as well as the length of the observation, since AKI occurrence is more common in the early phase of the hospitalization (see table). Our results suggest an AKI incidence of about 10%, and although it shows a low incidence, it aligns substantially with existing findings (see table).

Across all our models, we observed high negative predictive value (NPV) and low positive predictive value (PPV), which reflects the relatively low prevalence of positive cases in the dataset. Notably, sensitivity and specificity were reasonably well balanced  (0.72 and 0.70) in the model predicting AKI at admission (AKI_T0). These results are noteworthy, given the minimal data available at the time of that hospital admission (i.e. admission modality, pre-admission and admission laboratory values). However, the subsequent models predicting the transition to AKD (AKD_T1–T4) demonstrated improved sensitivity (from 0.79 to 0.94) and specificity (from 0.70 to 78), with performance increasing over time as more clinical data became available for prediction. Given their high NPV and sensitivity, the models could effectively rule out low-risk patients early in the hospital stay, helping prioritize monitoring and resource allocation. A high NPV is generally more desirable in the context of a low-prevalence disease.

We believe we have addressed all points and incorporated these comments into the discussion among the study limitations.

Table: studies investigating AKI prevalence in unselected subjects admitted to the hospital:

| Study | Total AKI Rate | Hospital-Acquired | Detection Rate | Refrence |
| --- | --- | --- | --- | --- |
| Bedford et al., 2014 | 15.3% of non-maternity and non-day case admissions | 73.6% on admission, 26.4% hospital-acquired | No mention found | M.Bedford, P. Stevens, Toby Wheeler, and C. Farmer. “What Is the Real Impact of Acute Kidney Injury?” BMC Nephrology, 2014. |
| Fang et al., 2010 | 3.19% | No mention found | No mention found | Yi Fang, Xiaoqiang Ding, Yi-hong Zhong, Jianzhou Zou, J. Teng, Ying Tang, Jing Lin, and Pan Lin. “Acute Kidney Injury in a Chinese Hospitalized Population.” Blood Purification, 2010 |
| Esposito et al., 2024 | 24.50% | No mention found | 32% (68% undetection rate) | Pasquale Esposito, F. Cappadona, M. Marengo, M. Fiorentino, Paolo Fabbrini, A. Quercia, Francesco Garzotto, Giuseppe Castellano, Vincenzo Cantaluppi, and F. Viazzi. “Recognition Patterns of Acute Kidney Injury in Hospitalized Patients.” Clinical Kidney Journal, 2024. |
| Wang et al., 2012 | 22.70% | No mention found | No mention found | Henry E Wang, Paul Muntner, Glenn M Chertow, David G Warnock. Acute kidney injury and mortality in hospitalized patients. Am J Nephrol . 2012;35(4):349-55. doi: 10.1159/000337487. Epub 2012 Apr 2 |
| Susantitaphong et al.,2013 | 21.6% in adults, 33.7% in children | No mention found | No mention found | P. Susantitaphong, D. Cruz, J. Cerdá, Maher M Abulfaraj, Fahad Alqahtani, Ioannis Koulouridis, and B. Jaber. “World Incidence of AKI: A Meta-Analysis.” American Society of Nephrology. Clinical Journal, 2013. |
| Challiner et al., 2014 | 25.40% | Approximately 1/3 on admission, 2/3 hospital-acquired | No mention found | R. Challiner, J. Ritchie, C. Fullwood, P. Loughnan, and A. Hutchison. “Incidence and Consequence of Acute Kidney Injury in Unselected Emergency Admissions to a Large Acute UK Hospital Trust.” BMC Nephrology, 2014. |
| Fenna et al., 2019 | 14.7% overall, 6.2% hospital-acquired | 8.5% community-acquired,6.2% hospital-acquired | No mention found | Kate Fenna, R. Erasmus, and A. Zemlin. “Hospital-Acquired Acute Kidney Injury Prevalence in in Adults at a South African Tertiary Hospital.” African Health Sciences, 2019. |
| Aitken et al., 2013 | 14.90% | 4.6% on admission, 10.3% hospital-acquired | 76.5% (23.5% unrecognized) | E. Aitken, C. Carruthers, L. Gall, L. Kerr, C. Geddes, and D. Kingsmore. “Acute Kidney Injury: Outcomes and Quality of Care.” QJM : Monthly Journal of the Association of Physicians, 2013. |
| Asmus et al., 2021 | 10.10% | No mention found | No mention found | K. Asmus, S. Erfurt, O. Ritter, S. Patschan, and D. Patschan. “AKI Epidemiology and Outcomes: A Retrospective Cohort Study from the Prenephrology Era.” International Journal of Nephrology, 2021. |
| Khadzhynov et al., 2019 | 21.40% | No mention found | 28.8% (71.2% not coded as AKI) | D. Khadzhynov, D. Schmidt, J. Hardt, G. Rauch, P. Gocke, K. Eckardt, and K. Schmidt-Ott. “The Incidence of Acute Kidney Injury and Associated Hospital Mortality.” Deutsches Ärzteblatt International, 2019. |

- Please address the points from Reviewer 3, in addition to further points from Reviewer 4 and if not possible include these limitations in the discussion.

**Answer**: We thank the reviewers for their thoughtful revision of the manuscript. We believe we have addressed all points (see below) and incorporated these comments into the discussion.

**Reviewer #1**: The authors performed the changes. They answered all the questions and modified the manuscript accordingly.

I have nothing to add. Good luck!

**Answer:** We thank the reviewer for the praise and the thoughtful revision of the manuscript

**Reviewer #2**: Dear Authors, I found your work interesting from the start, but I believe it has been significantly improved thanks to the revisions received and your subsequent modifications. I think this has been an excellent team effort. I have no further comments.

**Answer:** We thank the reviewer for the praise and the thoughtful revision of the manuscript.

**Reviewer #3:** The revision form of the study “usability of machine learning algorithms for the prediction of AKI and transition to AKD” of Ruinelli and co-authors still now hasn’t answered to the points previously underlined.

Mainly three aspects appear to be limiting.

**Answer:** We thank the reviewer for the thoughtful revision of the manuscript. Overall, we agree that this is a *proof of concept* study that warrants further confirmation in the future, prospective and appropriately designed studies. The main findings of the study indicate that the transition from AKI to AKD can be predicted by a simple machine learning (ML) model that utilizes data available in each patient’s chart. While this may raise awareness of AKD, if further validated, it may result in more patient-centered care.

The first is concerning the mathematical process of machine learning. The external validation consists on the testing of model in a different “external” group of patients, but in this case patients of external validation are those “internal” of hospital database. Therefore there isn’t an external validation. The external test is necessary to prove the model performance and its ability to generalize on different geographical populations.

(Ref. Alfieri et al, PLoS ONE 18(7): e0287398. <https://doi.org/10.1371/journal.pone.0287398>). The second point is the calibration test. When a curve is generated, a Brier Score can be computed, and a Bias detection can be made. This aspect is not treated in the present study

**Answer:** As highlighted among the study limitations, this is a retrospective *proof-of-concept* study, and current results warrant future validation. While external validation was not feasible (we don’t have another independent dataset to test the ML model performances), we adopted a standard approach of dividing the dataset into a training and a validation set.

While both the training and validation sets are subsets of the original dataset, they are independent. The training set is for learning, while the validation set evaluates the model's performance and mitigates issues like overfitting. Metrics such as accuracy, precision, recall, F1 score, and area under the ROC curve (AUC-ROC) are usually employed to evaluate the model's performance on the validation dataset.

While external validation offers insights into the model's robustness and reliability and, hence, on the potential generalizability and applicability of the ML algorithm in real-world scenarios, the methodology utilized in our study should ensure that the model performs well beyond the training dataset. This would open the venue for future studies to evaluate the model's generalizability and impact on patient management.

We have addressed all these points among the study's limitations, but we remain available for any further changes the reviewers feel are appropriate.

The third point is the very low Positive Predictive Value.

Regarding the low incidence of AKI in our cohort, we respectfully note that studies using established criteria typically report an AKI prevalence of 10-30% among unselected hospital admissions (see table). The wide range of reported AKI prevalence in admitted patients is likely due to various reasons, such as diagnostic criteria and administrative versus laboratory data used, as well as the length of the observation, since AKI occurrence is more common in the early phase of the hospitalization (see table). Our results suggest an AKI incidence of about 10%, and although it shows a low incidence, it aligns substantially with existing findings (see table).

A high negative predictive value (NPV) is generally more desirable in the context of a low-prevalence disease. NPV refers to the probability that individuals with a negative test result truly do not have the disease. When the prevalence of a disease is low, the number of true positives (people who have the disease) is also low. A high NPV means that most individuals who receive a negative result are indeed free of the disease, which is particularly important in low-prevalence scenarios to avoid unnecessary anxiety and further testing for patients. Conversely, a low NPV could result in a false sense of security, leading to undetected cases.

We have addressed all these points among the study's limitations, but we remain available for any further changes the reviewers feel are appropriate.

All these aspects deeply limit the study.

**Answer:** We agree with the reviewer that our study has limitations that we believe we have highlighted among the study's limitations. Still, we remain available for any further changes the reviewers feel are appropriate.

Reviewer #4: Some other comments
- Please verify the font size, as it appears inconsistent across the first three lines.
**Answer:** We thank the reviewer for the thoughtful revision of the manuscript. We have now ensured the consistency of the font throughout the manuscript.

- Was septic state considered in the model?

**Answer:** The septic state was indirectly incorporated into the model by using the laboratory data on C-reactive protein, white cell count, and emergency vs. elective hospital admission indirectly incorporated the septic state into the model.

- I still believe it is essential to describe a hypothetical sample size in order to indicate the statistical power of the ML results and their associated NPV, particularly given the low incidence of AKI and AKD, as mentioned by the authors.

**Answer:** We appreciate the referee's insightful comment regarding the necessity of describing a hypothetical sample size.

Power analysis can be calculated for both observational prospective cohort studies and randomized clinical trials. Although power can be computed *post hoc* using retrospective studies that employ Real-World Data (RWD), the "effect size" must be well-defined. In case series studies grounded in RWD, where data is collected longitudinally for monitoring purposes without a designated cohort or control sample, the "effect size" is not typically a primary focus.

The literature indicates that employing an estimated effect size to calculate post hoc power in observational studies can result in significant overestimations of power. Therefore, it is imperative to ensure that the dataset is both representative and sufficiently large to capture the variability of the outcome and predictor variables.

Furthermore, it is important to highlight that, in the realm of machine learning, a widely recognized rule of thumb suggests that a dataset should contain at least ten observations per covariate (i.e., ten cases per variable in the model) to mitigate the risk of overfitting and to guarantee stable model estimates. We are confident that our sample sizes are ample enough to support reliable model fitting and generalization, with the number of observations for each model ranging from 1,043 to 34,579. This robust performance should alleviate concerns regarding overfitting. This recommendation aligns with findings in the machine learning literature, which emphasize that flexible models typically necessitate considerably more data to preserve generalization performance.

Research focusing on health interventions has indicated that a minimum sample size of 500 to 1,000 is essential to mitigate overfitting and ensure reliable model performance. This range was suggested based on observed performance stabilization within these sample sizes.

While power analysis is a conventional practice in experimental studies, in the context of large retrospective datasets and machine learning models, the sheer size of the data often provides adequate power to effectively assess the relationships between variables and predict outcomes.

Regarding the low incidence of AKI in our cohort, we respectfully note that studies using established criteria typically report an AKI prevalence of 10-30% among unselected hospital admissions (see table). The wide range of reported AKI prevalence in admitted patients is likely due to various reasons, such as diagnostic criteria and administrative versus laboratory data used, as well as the length of the observation, since AKI occurrence is more common in the early phase of the hospitalization (see table). Our results suggest an AKI incidence of about 10%, and although it shows a low incidence, it aligns substantially with existing findings (see table).

A high negative predictive value (NPV) is generally more desirable in the context of a low-prevalence disease. NPV refers to the probability that individuals with a negative test result truly do not have the disease. When the prevalence of a disease is low, the number of true positives (people who have the disease) is also low. A high NPV means that most individuals who receive a negative result are indeed free of the disease, which is particularly important in low-prevalence scenarios to avoid unnecessary anxiety and further testing for patients. Conversely, a low NPV could result in a false sense of security, leading to undetected cases.

We have addressed all these points among the study's limitations, but we remain available for any further changes the reviewers feel are appropriate.

**References:**

[1] Gelman, A. Don't calculate post-hoc power using observed estimate of effect size. Ann. Surg, (2019) 269, e9-e10.

[2] Rajput, D., Wang, WJ. & Chen, CC. Evaluation of a decided sample size in machine learning applications. *BMC Bioinformatics* **24**, 48 (2023). <https://doi.org/10.1186/s12859-023-05156-9>

[3] Steyerberg, E. W., Vickers, A. J., Cook, N. R., Gerds, T. A., Obuchowski, N. A., & Pencina, M. J. (2010). Assessing the performance of prediction models: A framework for some traditional and novel measures. *Statistics in Medicine, 29*(21), 2619–2645. <https://doi.org/10.1002/sim.3835>

[4] Zantvoort, et al. K., Nacke, B., Görlich, D. *et al.* Estimation of minimal data sets sizes for machine learning predictions in digital mental health interventions. *npj Digit. Med.* **7**, 361 (2024).

- A possible consideration, in addition to the absence of concomitant therapies, could be the potential role of gender in the progression to AKI or AKD. Are the tools included in the model represented or describe even in supplementary?

**Answer:** We appreciate the referee's insightful comment regarding the potential role of gender in the progression of AKI to AKD. While we aimed to predict the AKI to AKD transition in the whole cohort and not to investigate any potential gender difference in outcome occurrence, gender was included in the ML models, and the SHAAP analysis does not suggest that gender is a predictive factor of the AKI to AKD transition. We believe that the suggested analyses are outside the scope of our analysis. We have included this point among the study limitations.

- As pointed out by the authors, the generalizability of the results remains debatable due to the lack of an external validation system for the ML model, and the cohort bias may be a significant factor influencing the results (low prevalence of AKI and AKD).

Despite these considerations, the work provides valuable insights and is well-structured and rigorous.

**Answer:** We thank the reviewer for the praise and the thoughtful revision of the manuscript.
